# An Enhanced Particle Swarm Optimization-Based Node Deployment and Coverage in Sensor Networks

**DOI:** 10.3390/s24196238

**Published:** 2024-09-26

**Authors:** Kondisetty Venkata Naga Aruna Bhargavi, Gottumukkala Partha Saradhi Varma, Indukuri Hemalatha, Ravilla Dilli

**Affiliations:** 1 Computer Science Engineering, Koneru Lakshmaiah Education Foundation, Hyderabad 500075, Telengana, India; bhargavi@klh.edu.in; 2 Computer Science Engineering, Koneru Lakshmaiah Education Foundation, Vijayawada 520002, Andhra Pradesh, India; gpsvarma@kluniversity.in; 3 Information Technology, SRKR Engineering College, Bhimavaram 534204, Andhra Pradesh, India; drihl@srkrec.ac.in; 4 Electronics and Communication Engineering, Manipal Institute of Technology, Manipal Academy of Higher Education, Manipal, Udupi 576104, Karnataka, India

**Keywords:** coverage problem, Delaunay triangulation, particle swarm optimization, sensor node deployment, wireless sensor network

## Abstract

Positioning, coverage, and connectivity play important roles in next-generation wireless network applications. The coverage in a wireless sensor network (WSN) is a measure of how effectively a region of interest (ROI) is monitored and targets are detected by the sensor nodes. The random deployment of sensor nodes results in poor coverage in WSNs. Additionally, battery depletion at the sensor nodes creates coverage holes in the ROI and affects network coverage. To enhance the coverage, determining the optimal position of the sensor nodes in the ROI is essential. The objective of this study is to define the optimal locations of sensor nodes prior to their deployment in the given network terrain and to increase the coverage area using the proposed version of an enhanced particle swarm optimization (EPSO) algorithm for different frequency bands. The EPSO algorithm avoids the deployment of sensor nodes in close proximity to each other and ensures that every target is covered by at least one sensor node. It applies a probabilistic coverage model based on the Euclidean distances to detect the coverage holes in the initial deployment of sensor nodes and guarantees a higher coverage probability. Delaunay triangulation (DT) helps to enhance the coverage of a given network terrain in the presence of targets. The combination of EPSO and DT is applied to cover the holes and optimize the position of the remaining sensor nodes in the WSN. The fitness function of the EPSO algorithm yielded converged results with the average number of iterations of 78, 82, and 80 at 3.6 GHz, 26 GHz, and 38 GHz frequency bands, respectively. The results of the sensor deployment and coverage showed that the required coverage conditions were met with a communication radius of 4 m compared with 6–120 m with the existing works.

## 1. Introduction

Wireless sensor networks (WSNs) play a vital role in the perception layer of Internet of Things (IoT) network architectures and support smart capabilities in the automation of homes, agriculture, cities, electric grids, etc. Since WSNs are composed of highly resource-constrained devices, challenges such as power optimization, node deployment, network lifetime, connectivity, and coverage exist that need to be addressed. The practical deployment of WSNs has multiple objectives, such as energy efficiency, resource allocation, connectivity, and coverage, which are conflicting in nature. The optimization of sensor node (SN) deployment is very challenging in such scenarios. In WSNs, the random distribution of sensor nodes causes low connectivity and coverage rates; therefore, computing the optimal locations for sensor node deployment is highly challenging because of the robustness of sensor node failures. Additionally, SN distribution and coverage optimization are highly important for enhancing the lifetime of WSNs. The coverage depends on the communication range, sensitivity, location, and density of the sensor nodes [1].

SNs must be deployed to cover a set of predetermined locations called “targets” in the region of interest (ROI). The coverage model in WSNs represents the sensing capabilities of the SNs within the ROI and predicts the probability of covering the targets. The selection of a coverage model in a WSN depends on various factors, including the SN mobility, ROI location, network size, node density, and deployment application. In this study, we propose an enhanced version of particle swarm optimization (PSO) to address node deployment and coverage problems in WSNs.

PSO is one of the most popular swarm intelligence and population-based optimization algorithms for solving optimization problems in various applications. This approach is inspired by the social behavior of birds and fish flocks searching for food, shelter, etc. Individual particles in a swarm move to suitable locations based on their adaptation to the environment. PSO uses multiple particles to form a swarm, and each particle moves around in the search space to find the best solution. Each particle in the swarm flies in the search area, and the best solution to be positioned is determined. Each particle is a candidate solution, and the sets of candidate solutions coexist simultaneously in a cooperative manner. The position of each particle is updated based on the best solution from its neighbors along with its own best-known solution.

The conventional PSO algorithm generates a swarm of ‘P’ particles and handles this population as N-dimensional vectors. Therefore, it is challenging to maximize the fitness function when higher-dimensional vectors are used as inputs. The proposed enhanced PSO (EPSO) algorithm generates ‘N’ one-dimensional swarms, and each swarm consists of ‘P’ particles. In EPSO, each particle in a swarm is one-dimensional, and each swarm attempts to optimize an element in the solution vector.

The current research presented in this paper focuses on SN deployment and coverage problems in planar WSNs. Existing empirical studies [2,3,4] have shown their ability to provide optimal solutions with smaller swarm sizes of 10 to 50 particles at a higher number of iterations, between 300 and 3000, and the sensor node communication range is greater, ranging from 6 m to 40 m. In addition, the conventional PSO algorithm has limitations, including a lower convergence rate, premature convergence, and a local optimal solution when the search space is high-dimensional. To overcome these limitations, EPSO is proposed in this study, which provides a global optimal solution for placing SNs. We aimed at obtaining higher swarm sizes of up to 100 and applying them to optimize the SN locations and coverage in WSNs. The battery depletion levels of the SNs are considered while calculating the node locations, and the communication range is minimized to improve the overall lifetime of the network. It is based on the Euclidean distance of each SN with respect to the center of the network terrain.

The main contributions include the following:

(i) The EPSO algorithm is proposed to avoid the deployment of SNs in proximity to each other, and it also considers the battery lifetime of the SNs to increase the network lifetime.
(ii) A simple coverage model is defined for deploying SNs, such that every target is covered by at least one SN.
(iii) A probabilistic coverage model based on Euclidean distances is applied to detect coverage holes during the initial deployment of SNs and ensure higher coverage probability.
(iv) The method considers the position of the SN when defining the objective function and minimizes the computational complexity, as it generates ‘N’ one-dimensional swarms instead of N-dimensional swarms.
(v) The appropriate inertia weights of the particle and acceleration factors are defined when optimizing the objective function. This function is applied for tuning the SN positions to cover holes and enhance the network coverage.
(vi) The EPSO algorithm, along with Delaunay triangulation (DT), is applied to cover the holes and enhance the coverage of a given network terrain in the presence of targets, and the algorithm yields converged results after a lesser number of iterations over the existing techniques.

The remainder of this article is organized as follows: Section 2 presents the nascent works on node deployment and coverage strategies in WSNs; Section 3 provides the details of the EPSO algorithm and its pseudocode; Section 4 discusses the details of simulation specifications and results; and Section 5 concludes the current work and discusses possible future directions.

## 2. Existing Works

In this section, the features of state-of-the-art node deployment and coverage algorithms in WSNs are well studied, and mainly PSO-based node deployment and coverage techniques are listed in Table 1. Optimal coverage through a “binary ant colony optimization” algorithm is proposed for WSNs, and the inclusion of additional search mechanisms, known as “hill climbing” and “simulated annealing”, further refines the optimal solution for coverage in WSNs. The additional capabilities enhance the exploration of the search space and provide a superior configuration of SN placement for the best coverage [5]. The combination of cuckoo search, particle swarm, and opposition-based learning can enhance the range coverage in WSNs by converting regional monitoring into point monitoring. It yields 82.58% and 97.44% coverage efficiency for SN counts of 20 and 30, respectively [6]. To support the strong connectivity of SNs in harsh environmental applications, an optimal deployment strategy is introduced using “double-state differential evolution”, which is a metaheuristic approach. It has shown favorable performance in 2D space. The dynamic network concept allows connectivity updates of newly entered nodes and avoids recomputing the whole network’s connectivity status [7]. The “decomposition-based evolutionary algorithm” is proposed to address the challenges in a multi-objective deployment scenario of WSNs. Seven objectives with certain weights are considered to support SN deployment at the cost of 314 sec of processing time [8]. An “intelligent satin bower bird optimizer” with reinforcement learning capabilities can enhance the connectivity and coverage of SNs in WSNs. SNs are adapted to the variable conditions and performance of the network. Eight anchor nodes and nineteen SNs are used to detect seventy-five target nodes [9]. Different classes of targets (i.e., targets belonging to different domains) are covered based on disjoint sensor deployment to enhance coverage with minimal power consumption [10]. The “convex optimization” and “integer programming” techniques used in SN deployment consider the trade-off between power consumption and interference, and introducing a metaheuristic approach enhances scalability [11]. Greedy metaheuristic approaches determine the optimal locations for SN deployment for target coverage while ensuring lifetime optimization in WSNs. The work presented in [12] showed that the network lifetime is enhanced by 125–269%. The fitness of a “firefly optimization” algorithm is designed considering multiple parameters such as node survivability, communication distance, connectivity, number of nodes, and coverage. SNs satisfying q-connectivity and target nodes satisfying p-coverage provide the optimal SN deployment [13]. Energy-efficient covering strategies using the “grey wolf optimization” algorithm were proposed in [14], which mainly address optimal SN deployment in WSNs. SN deployment is further challenging in underwater WSNs due to the node mobility caused by water currents and communication delays [15]. An improved “sparrow search” algorithm proposed in [16] improves the node connectivity and coverage by 11.8% and 8.4%, respectively, in meeting the optimal deployment requirements of underwater WSNs. The “salpa swarm intelligence” algorithm aims to improve node coverage and minimize node deployment costs and energy consumption in WSNs [17]. The network coverage ranged from 80% to 95% when 27 to 41 SNs were used.

The literature on the aspects of SN deployment and coverage, particularly based on PSO, has maintained higher communication ranges that deplete the SN’s battery faster. Additionally, smaller swarm sizes are considered when defining the optimal solutions. Few of the existing algorithms [40,41,42] based on improved versions of PSO define the objective function as an N-dimensional swarm, which leads to increased computational complexity. In high-dimensional space, these algorithms lead to premature convergence and provide a local optimal solution.

To overcome these limitations, an enhanced version of PSO is proposed in this study with a refined fitness function, which provides a global optimal solution for placing SNs. We aimed at obtaining swarm sizes of up to 100 and applied them to optimize the SN locations and coverage in WSNs. The communication range of the SNs is minimized to save the battery power levels and further improve the overall network lifetime. It is based on the Euclidean distance of each SN with respect to the center of the network terrain.

The method considers the position of the SN when defining the objective function and minimizes the computational complexity, as it generates ‘N’ one-dimensional swarms instead of N-dimensional swarms. The appropriate inertia weight of the particle and acceleration factor are defined when optimizing the objective function. This function is applied for tuning the SN positions to cover holes and enhance the network coverage.

## 3. Materials and Methods

There are multiple ways of defining the sensor deployment problem: *simple coverage* defines the SN deployment such that every target is covered by at least one SN. *K-coverage* defines SN deployment in the given sensing area such that every target is covered by at least ‘k’ SNs. *Q-coverage* considers a vector $q=\left\{q_{1},q_{2},\;q_{3},\;\ldots \ldots \ldots ,q_{k}\right\}$, deploying SNs such that each target is covered by at least $q_{k}$ SNs.

Let ‘S’ be the total number of SNs deployed in a 100 m × 100 m two-dimensional network terrain at locations $\left\{\left(x_{1},y_{1}\right),\;\left(x_{2},y_{2}\right),\;\left(x_{3},y_{3}\right),\;\ldots \ldots \ldots \;\left(x_{S},y_{S}\right)\right\}$. To avoid dimension dependency, the EPSO algorithm considers one-dimensional ‘S’ swarms instead of a single S-dimensional swarm. Each one-dimensional swarm is a set of feasible SN deployment locations in a given network terrain. This makes the deployments of any two SNs independent of each other. The objective of the EPSO algorithm is to avoid the deployment of SNs closer to each other and to enhance the network lifetime.

Let each swarm consist of ‘P’ SNs located at $\left\{\left(x_{1},y_{1}\right),\left(x_{2},y_{2}\right),\left(x_{3},y_{3}\right),\ldots \ldots \ldots \left(x_{P},y_{P}\right)\right\}$, and the coverage vector contains ‘S’ pairs as $\left\{\left(x_{1},y_{1}\right),\left(x_{2},y_{2}\right),\left(x_{3},y_{3}\right),\ldots \ldots \ldots \left(x_{S},y_{S}\right)\right\}$, where $\left(x_{i},y_{i}\right)$ is the location of the $i^{th}$ SNs with 0$\;\leq x_{i}\leq$ 100 m, 0$\;\leq y_{i}\leq$ 100 m, and P < S.

**Figure 1:**
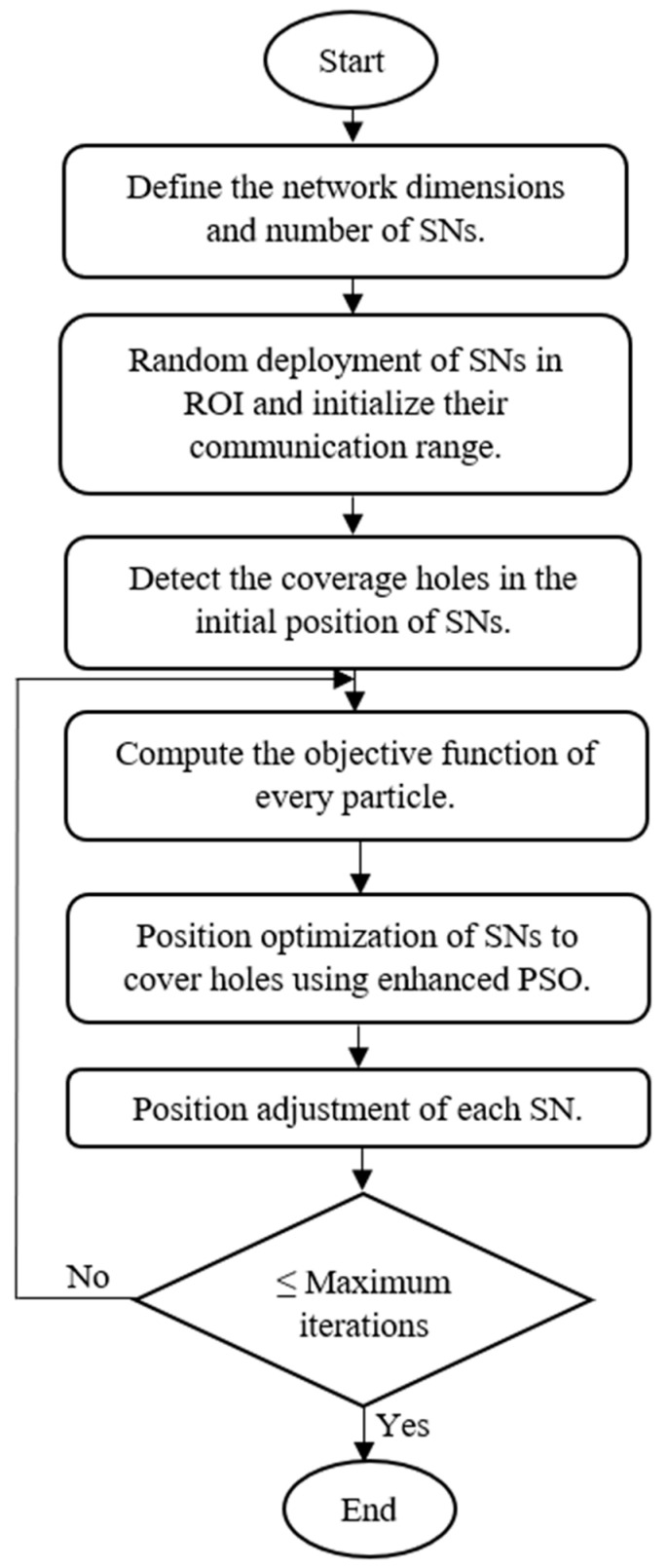
Flowchart of the proposed EPSO algorithm.

The flowchart of the proposed EPSO algorithm is shown in Figure 1.

1. The size of the sensor network is defined in terms of its dimensions and the number of SNs.
2. As part of initialization, the SNs are deployed randomly in the ROI such that every target is monitored in at least one SN and its communication range is initialized.
3. By following the *probabilistic* coverage model, we can detect coverage holes in the initial position of the SNs.
4. The objective function of every SN is calculated with the help of its position value.
5. The positions of the SNs are optimized and tuned to cover holes via EPSO.
6. Repeat steps 4 and 5 until the maximum number of iterations is reached.

Consider ‘S’ sensor nodes $\left\{N_{1},\;N_{2},N_{3},\;\ldots \ldots .N_{S}\right\}$, each with $E_{0}$ initial energy milliwatts, ‘r’ as the sensing radius in meters, and ‘T’ targets $\left\{M_{1},\;M_{2},M_{3},\;\ldots \ldots .M_{T}\right\}$ located in the (x, y) area. Any given sensor node $N_{j}$ covers the target $M_{k}$ if and only if $d_{N_{j}M_{k}}\leq r$. Accordingly, the coverage matrix [39] is defined using Equation (1), where $d_{N_{j}M_{k}}$ is the distance between sensor node $N_{j}$ and target node $M_{k}$.

(1) $$Coverage\;value,\;C_{jk}=\left\{\begin{array}{c} 1\;if\;d_{N_{j}M_{k}}\leq r\\ 0\;otherwise \end{array}\right.\mathrm{where}\;1\leq j\leq S,1\leq k\leq T$$

### 3.1. Battery Depletion Model

Multiple SNs may monitor the same ROI and cause energy waste. The failure of SNs due to battery depletion results in coverage holes in the ROI. The combination of the EPSO and DT algorithms provides energy-efficient coverage of the ROI. The optimal sleep patterns for redundant SNs are defined based on the node failure probability, coverage overlap, and neighboring SN battery discharge rate [35].

(2) $${Back-off\;sleep\;time,\;T}_{back-off}=\left(1-p\right)\times \left(T_{full}-T_{present}\right)$$

The back-off sleep time of an SN is defined using Equation (2), and it is measured in seconds, where ‘p’ is the SN failure probability, $T_{full}$ is the time needed to completely discharge the node’s battery in seconds, and $T_{present}$ in seconds is the time elapsed since the SN has been ON.

The prediction of battery charge depletion based on received signal strength indicator (RSSI) values has advantages because it can be directly read from the SN’s operating system. Therefore, in the present work, the first order “autoregressive” model is presented in Equation (3), which works based on RSSI measurements and is used to represent the battery depletion at SNs. It represents the charge depletion process, gives the absolute mean residue, and allows us to predict the behavior of the battery.

(3) $${RSSI}_{t}\left(in\;dBm\right)=a_{1}{\times RSSI}_{t-1}+N_{t}$$

$a_{1}$ is a first-order autoregressive model parameter [36], and $N_{t}$ is the white Gaussian noise whose standard deviation is 0.1 dBm. Accordingly, the sensor network lifetime is defined as the fitness function and is computed in terms of seconds as follows:

(4) $$Network\;lifetime,\;T=\begin{array}{c} min\\ k \end{array}\frac{\sum_{j}^{S}C_{jk\;}}{q}\;\left(\frac{P_{j}}{E_{j}}\right)$$

In Equation (4), q’ is the coverage factor, and the second term, $\left(\frac{P_{j}}{E_{j}}\right)$, defines the SN’s battery lifetime; it is described as follows:

$$Battery\;lifetime\;of\;sensor\;node\;‘j’=\frac{Initial\;battery\;power,\;P_{j}}{Energy\;consumption\;rate,\;E_{j}}$$

The upper bound of the network lifetime is measured in seconds, and it is represented by Equation (5).

(5) $${Upper\;bound\;of\;network\;lifetime,\;L}_{up}=\frac{\sum_{j}C_{jk}\times Battery\;lifetime}{q_{k}}$$

where $q_{k}=k$ for k-coverage.

The sensitivity of any given ${SN}_{n}$ toward the target $T_{i}$ is represented by Equation (6).

(6) $$\mathrm{Sensitivity},\;S\left({SN}_{n},T_{i}\right)=\frac{\alpha }{{\left[d\left({SN}_{n},T_{i}\right)\right]}^{l}}$$

α and l are positive constants that depend on the SNs. $d\left({SN}_{n},T_{i}\right)$ are the Euclidean distances between the given SN and the target, whose coordinates are $\left(x_{n},y_{n}\right)$ and $\left(x_{i},y_{i}\right)$, respectively.

The most effective and popular coverage models for WSNs are broadly classified as *binary* or *probabilistic*. The operation and performance of existing coverage models in the literature lie between those of *binary* and *probabilistic* models. In the *binary* coverage model, the coverage probability is 100% if the Euclidean distance between the target node and SN is less than the sensing radius; otherwise, the coverage probability is zero. This model is applicable for ideal scenarios and does not consider the built-in inaccuracies of SNs, environmental conditions, interference due to external sources, or the presence of obstacles in the ROI. The *probabilistic* coverage model addresses these limitations and considers the uncertainty involved in sensing the targets. The coverage probability values of the *binary* and *probabilistic* models are given by Equation (7).

(7a) $$C_{xy}\left({SN}_{n}\right)=\left\{\begin{array}{c} 1,\;for\;r_{c}>d\left({SN}_{n},T_{i}\right)\;\\ 0,\;otherwise\; \end{array}\right.$$

(7b) $$C_{xy}\left({SN}_{n}\right)=\left\{\begin{array}{c} 1,\;for\;d\left({SN}_{n},T_{i}\right)\leq r_{c}-r_{u}\;\\ e^{\begin{array}{c} -\alpha k^{\beta }\\ \; \end{array}},\;for\;r_{c}-r_{u}<d\left({SN}_{n},T_{i}\right)\leq \;r_{c}+r_{u}\\ 0,\;for\;d\left({SN}_{n},T_{i}\right)>r_{c}+r_{u} \end{array}\right.$$

where α= $d\left({SN}_{n},T_{i}\right)-r_{c}-r_{u}$.

$r_{c}$ and $r_{u}$ are the communication radius in meters and sensing uncertainty, respectively, of a given sensor node ${SN}_{n}$. α and β are the values of sensing probability.

The position and velocity of the $P^{th}$ particle in an N-dimensional space are represented by vectors $S_{P}=\left(s_{P1},\;s_{P2},\;s_{P3},\ldots \ldots \ldots .,\;s_{PN}\right)$ and $V_{P}=\left(v_{P1},\;v_{P2},\;v_{P3},\ldots \ldots \ldots .,\;v_{PN}\right)$, respectively. The best positions observed by the $P^{th}$ particle and the complete swarm are vectors $B_{P}=\left(b_{P1},\;b_{P2},\;b_{P3},\ldots \ldots \ldots .,\;b_{PN}\right)$ and $B_{G}=\left(g_{1},\;g_{2},\;g_{3},\ldots \ldots \ldots .,\;g_{N}\right)$, respectively. Each particle is a solution to the optimization problem and is computed using a fitness function. To obtain the best solution, each particle in the swarm updates its position and velocity at every iteration using its own best solution as well as the best solution of the swarm. At each iteration, $B_{G}$ represents the best feasible location values for sensor deployment, and these values are placed in the coverage vector. The elements of $B_{G}$ are updated by a particle whose fitness value is maximum.

The proposed EPSO algorithm generates ‘N’ one-dimensional swarms, and each swarm consists of ‘P’ particles. In EPSO, each particle in a swarm is one-dimensional, and each swarm attempts to optimize an element in the solution vector. Since each swarm is one-dimensional, the objective function is computed using the “coverage vector.” The $i^{th}$ element corresponds to the desired particle, and while computing the $i^{th}$ element of the coverage vector, the remaining (N – 1) elements are kept constant. For each particle in the $i^{th}$ swarm, the coverage vector is defined such that, except for the $i^{th}$ element, all the elements correspond to $B_{G}$ vector elements. These coverage vectors are used to compute the fitness function.

At each iteration, the velocity and position vectors are updated using Equations (8) and (9), respectively.

(8) $$\mathrm{Velocity}\;\mathrm{vector},\;V_{p,n}\left(t+1\right)=w\times V_{p,n}\left(t\right)+a_{1}\times l\times (b_{p,\;n}─S_{p,\;n}\left(t\right))+a_{2}\times m\times (g_{n}─S_{p,\;n}\left(t\right))$$

(9) $$\mathrm{Position}\;\mathrm{vector},\;S_{p,n}\left(t+1\right)=S_{p,n}\left(t\right)+V_{p,n}\left(t+1\right)$$

where ‘w’ is the inertia weight of the particle, $a_{1}$ and $a_{2}$ are the acceleration factors that are nonnegative constants, l and m are uniformly distributed random numbers in the range [0,1], and $b_{p,\;n}$ and $g_{n}$ are the particle’s best position and global best position of the $n^{th}$ SN, respectively. The values of Equations (8) and (9) are updated in each iteration until the best values $B_{G}$ are achieved or the maximum iterations are reached. The first, second, and third terms in Equation (8) are the inertia, cognitive, and social parts, respectively.

The inputs to the EPSO algorithm include the number of swarms, number of particles in each swarm, and dimensions of the network terrain. Each particle in the swarm is randomly assigned location coordinates whose values are in the range 0≤x≤X and 0≤y≤Y. In the next step, the fitness function is computed using Equation (4) to maximize the sensor network lifetime. The velocity and position of each SN are updated at each iteration using Equations (8) and (9), respectively. If the newly computed location values are negative or out of range, they are updated using the following conditions: Generate new random location values when the (x, y) coordinates have negative values. Then, x ← X, y ← Y is assigned if the (x, y) coordinate values are beyond the network dimensions. Once the new location of the SN is computed, the corresponding element in the coverage vector is updated if and only if its fitness value is higher than the existing value. This process continues until the number of iterations reaches its maximum. Finally, the complete swarm vector $B_{G}$ represents all the SNs’ final locations that are optimal for enhancing the network lifetime as shown in Algorithm 1.
**Algorithm 1:** Pseudocode of the proposed EPSO algorithmInput:   S: Number of swarms;   P: number of particles in each swarm;   X × Y: dimensions of network terrain; Initialization:   Define the initial locations of ‘P’; $\mathrm{While}\;(i\leq \;t_{max})$      $/*t_{max}$: maximum number of iterations  For i=1 to S   For j=1 to P    Update the coverage vector;    $\mathrm{Compute}\;\mathrm{network}\;\mathrm{lifetime}\;T_{new}$ using Equation (4);    $\mathrm{If}\;B_{P}<T_{new}$ then     $B_{P}\;\leftarrow T_{new}$;    End if;     End for;  End for; $\mathrm{Compute}\;B_{G}\;\mathrm{from}\;\mathrm{all}\;\mathrm{particles}\;B_{P}$; $\mathrm{Coverage}\;\mathrm{vector}\;\leftarrow B_{G}$; For j = 1 to P Compute the particle velocity using Equation (8); Compute the particle position using Equation (9); End for; End While; $\mathrm{Output}:\;\mathrm{complete}\;\mathrm{swarm}\;\mathrm{vector},\;B_{G}$;

### 3.2. Delaunay Triangulation (DT)

**Figure 2:**
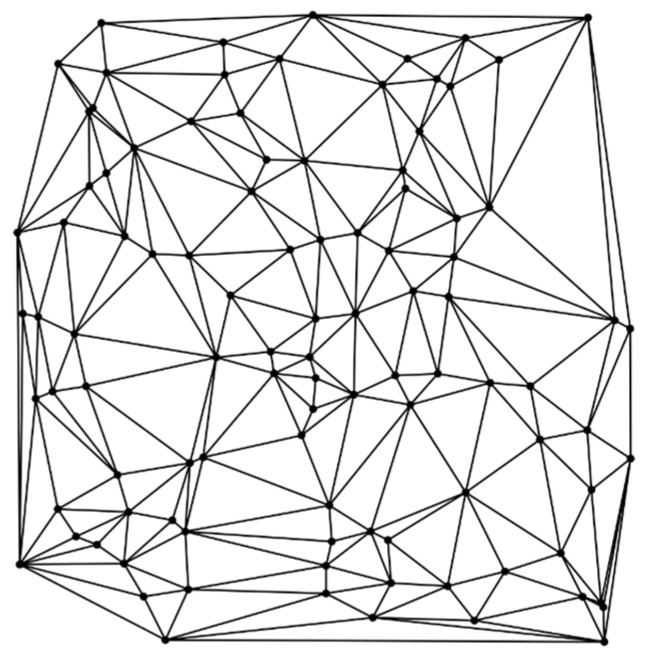
Delaunay triangulation in SN deployment [27].

A DT is applied to create a graph of edges between the SNs and to segment the network terrain into triangles, as shown in Figure 2.

*Steps to perform DT:* 

1. Create random points to triangulate.
2. Create a super triangle.
3. Compute the circumcircle.
4. Create new triangles.
5. Compute circumcircles of the new triangles.
6. Determine circumcircles that contain points.
7. Invalidate those triangles; they need to be replaced by smaller triangles.
8. Create new triangles.
9. Repeat the procedure.
10. Remove triangles that share an edge or vertex with the original super triangle.

The deployment of SNs is performed in the sparse region of the ROI based on the “empty circle property” of the DT. This approach avoids coverage holes at the boundaries of the ROI and enhances the coverage. The circumcircles of triangles without sensors are identified, and SNs are deployed at the center of the circumcircles to increase the coverage gain while supporting a minimal number of SNs.

First, the circumcenter of each triangle is computed, and the adjacent triangle common-side distance is subsequently calculated to detect holes. A DT as shown in Algorithm 2 helps to enhance the coverage of a given network terrain in the presence of targets. A probabilistic sensor model is used to compute the coverage gain, as shown in Equation (7a), and to avoid overlapping the sensing regions. The proposed EPSO algorithm, along with DT, is applied to cover the holes and optimize the position of the remaining SNs in the WSN.
**Algorithm 2:** Pseudocode of the DT algorithmInput:   DP: Number of discrete points; Initialization:   Triangulation ← Empty Triangle mesh data structure; Triangulation ← Super Triangle; // It must be large enough to contain all the discrete points. For p = 1 to DP       // add DP to Triangulation   Bad Triangles ← Empty set;   For each triangle in Triangulation do   // Find Triangles no longer valid due to insertion    If p is inside circumcircle of Triangle      BadTriangles ← Triangle;     Polygon ← empty set; For each Triangle in BadTriangles do    // Determine the boundary of polygonal hole   For each edge in Trianlge do    If edge is not shared by any other Triangle in BadTriangles      Polygon ← edge; For each Triangle in BadTriangles do    Remove Triangle from Triangulation; For each edge in Polygon do     // Re-Triangulate the polygonal hole      NewTriangle ← form a Triangle from edge to point;      Triangulation ← NewTriangle; For each triangle in triangulation do  If Triangle contains a vertex from original super Triangle    Remove Triangle from Triangulation; Return Triangulation;

## 4. Results and Discussion

**Figure 3:**
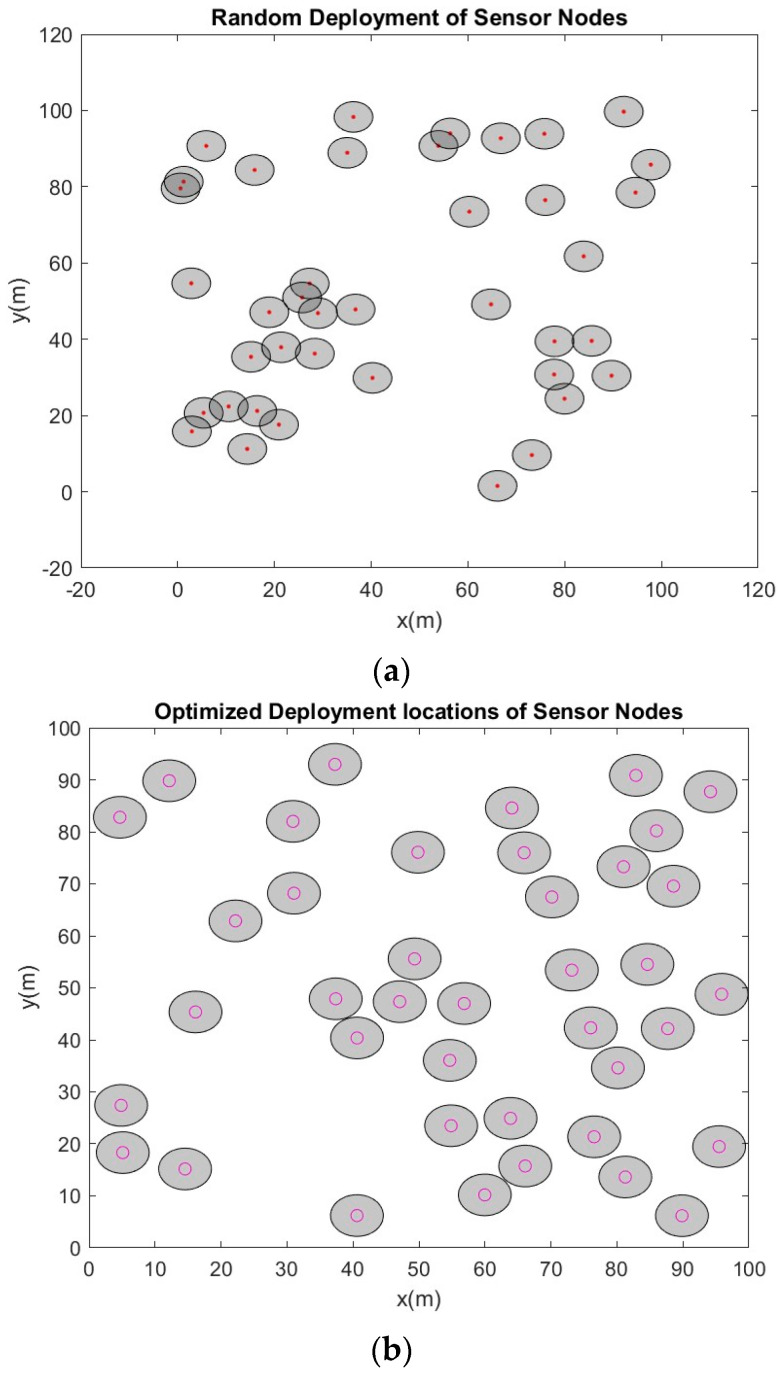

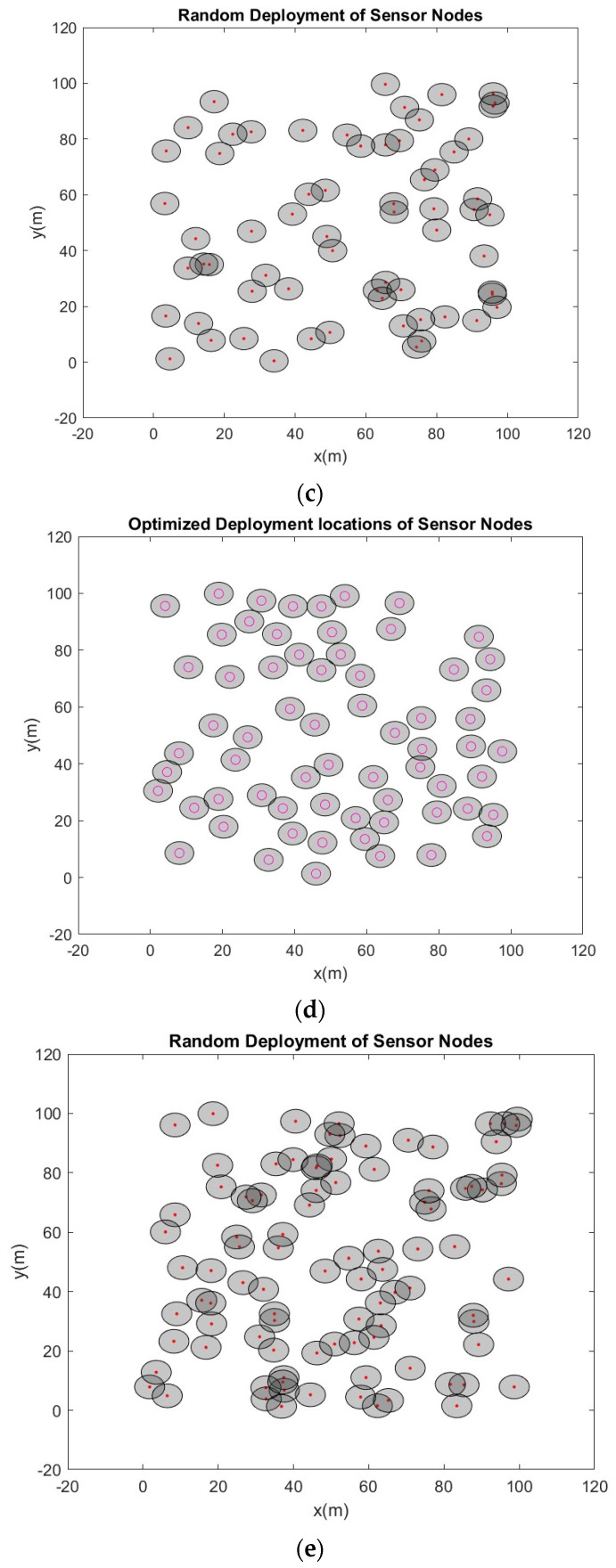

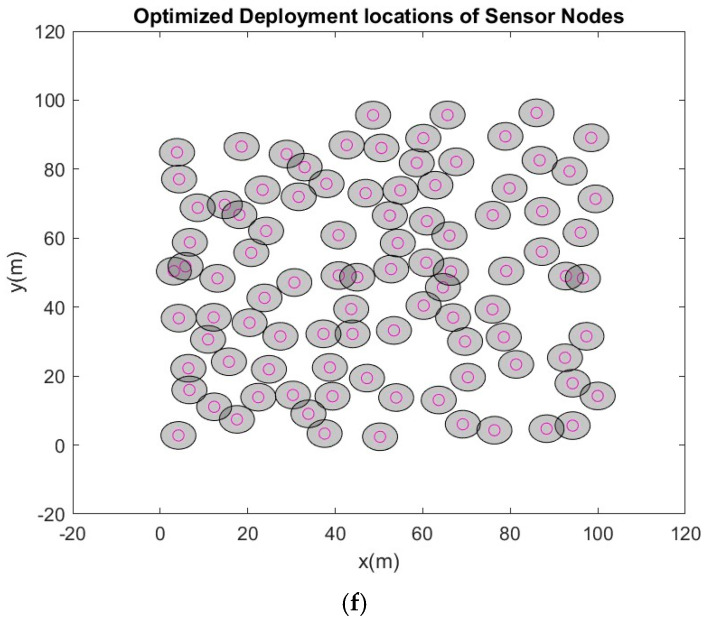
Initial locations of sensor nodes and their optimization using the proposed EPSO algorithm. (**a**) With 40 sensor nodes: initial locations of random deployment at 3.6 GHz. (**b**) With 40 sensor nodes: Optimized locations after applying the EPSO algorithm at 3.6 GHz. (**c**) With 65 sensor nodes: Initial locations of random deployment at 3.6 GHz. (**d**) With 65 sensor nodes: Optimized locations after applying the EPSO algorithm at 3.6 GHz. (**e**) With 95 sensor nodes: Initial locations of random deployment at 3.6 GHz. (**f**) With 95 sensor nodes: Optimized locations after applying the EPSO algorithm at 3.6 GHz.

**Figure 4:**
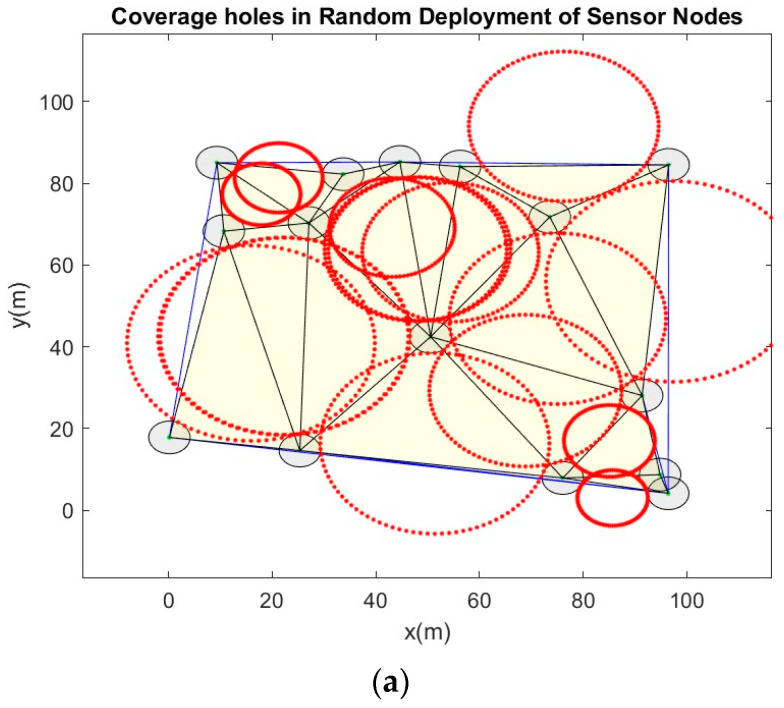

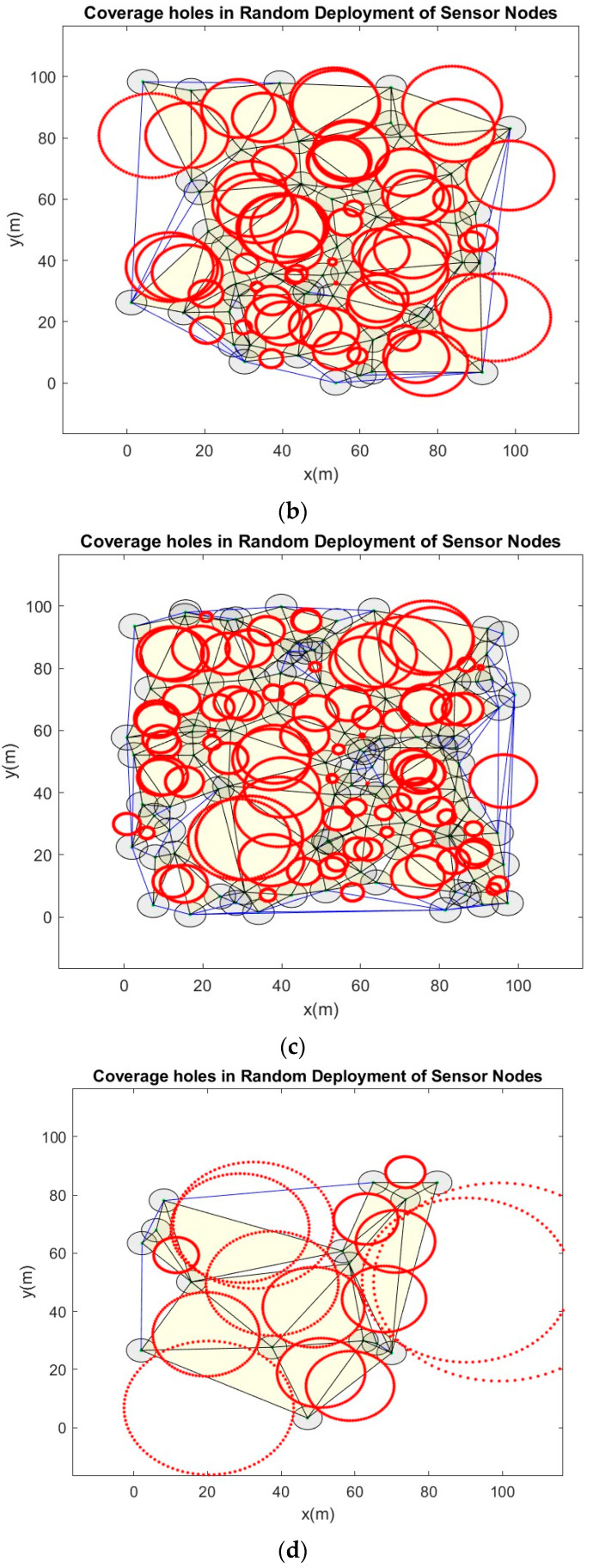

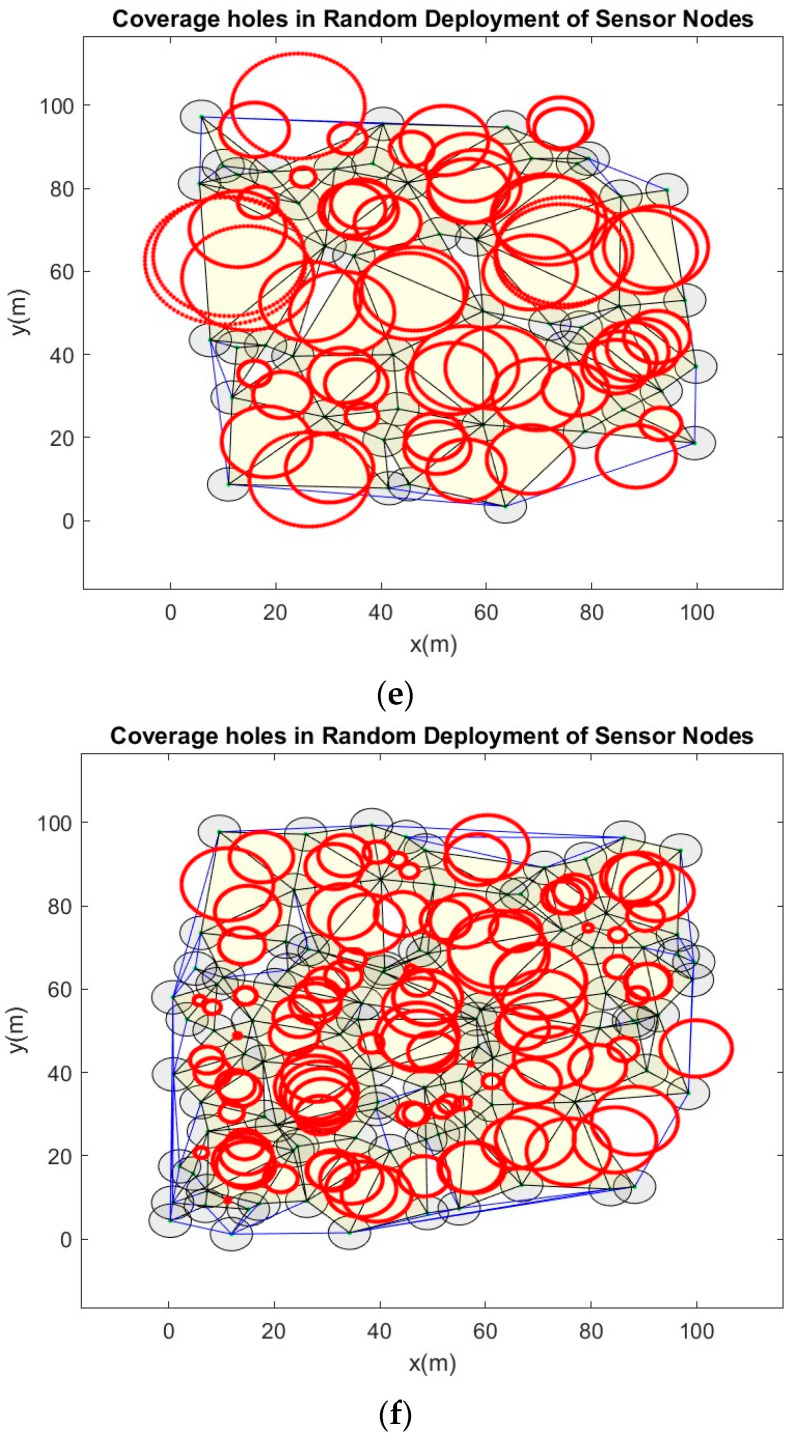
Coverage holes in random deployment of sensor nodes. (**a**) 15 sensor nodes at carrier = 26 GHz. (**b**) 45 sensor nodes at carrier = 26 GHz. (**c**) 90 sensor nodes at carrier = 26 GHz. (**d**) 15 sensor nodes at carrier = 38 GHz. (**e**) 45 sensor nodes at carrier = 38 GHz. (**f**) 90 sensor nodes at carrier = 38 GHz.

The performance of the proposed EPSO algorithm was verified through simulations and visualization of the results in a MATLAB 2024a environment. Simulations were conducted using an Office 365 operating system for an increased network size from ten to one hundred SNs in steps of five, and the other simulation parameters are listed in Table 2. SNs are assumed to be mobile and homogeneous in terms of their features. After their random deployment in the given network terrain, they were configured with a uniform radio range; their initial positions and nodes were assumed to be at their initial best local positions. The communication range of the SNs was set to twice their sensing radius. Figure 3 shows the results of random deployment versus optimal deployment for a few scenarios. Similarly, the simulations were conducted for different frequency bands in the FR1 and FR2 ranges (n48, n258, and n260 bands) [43]. The results of optimal deployment clearly showed that the presence of SNs closer to each other is avoided to the maximum extent. Then, coverage holes are detected by applying the DT method for the initial deployment of the SNs, as shown in Figure 4. Parameters such as the inertia weight and acceleration factors of the velocity vector and the battery depletion levels were optimally selected based on rigorous simulations and analysis of the results. The objective function was computed for each SN and the global best value was defined. The best value of the fitness function was used to derive the optimal locations of the SNs and to cover the detected holes.

**Figure 5:**
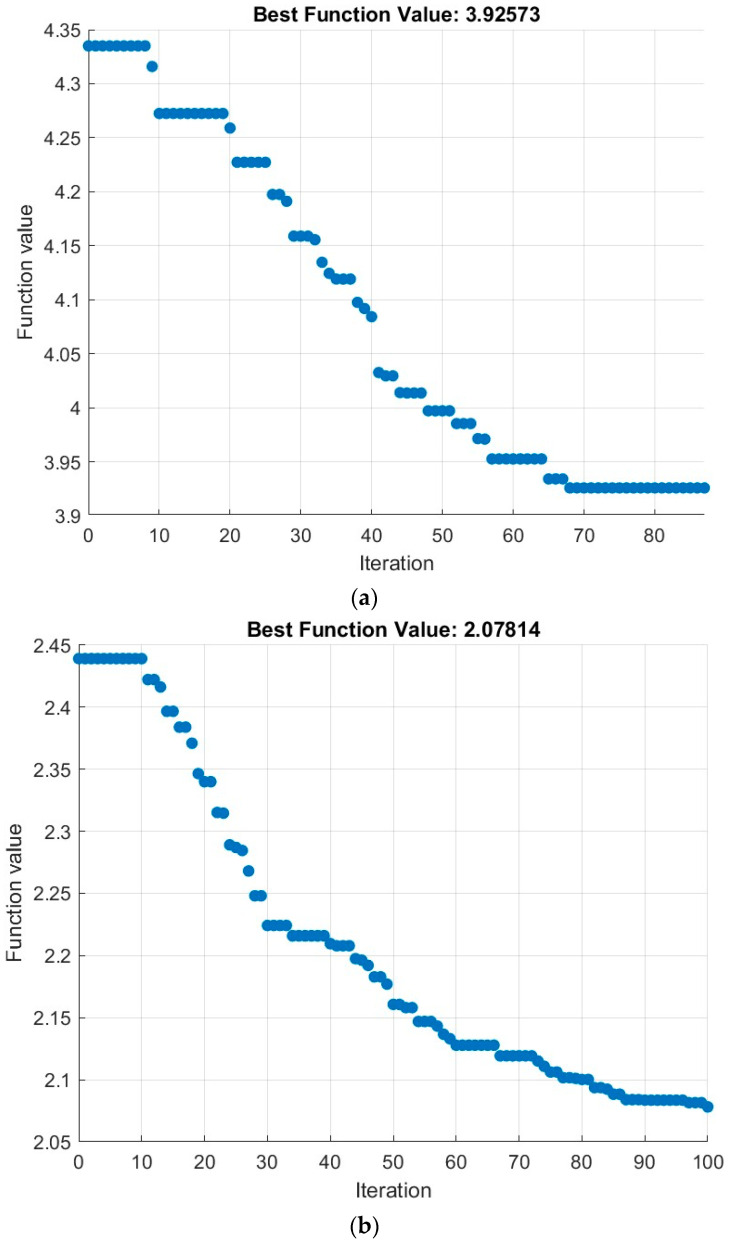

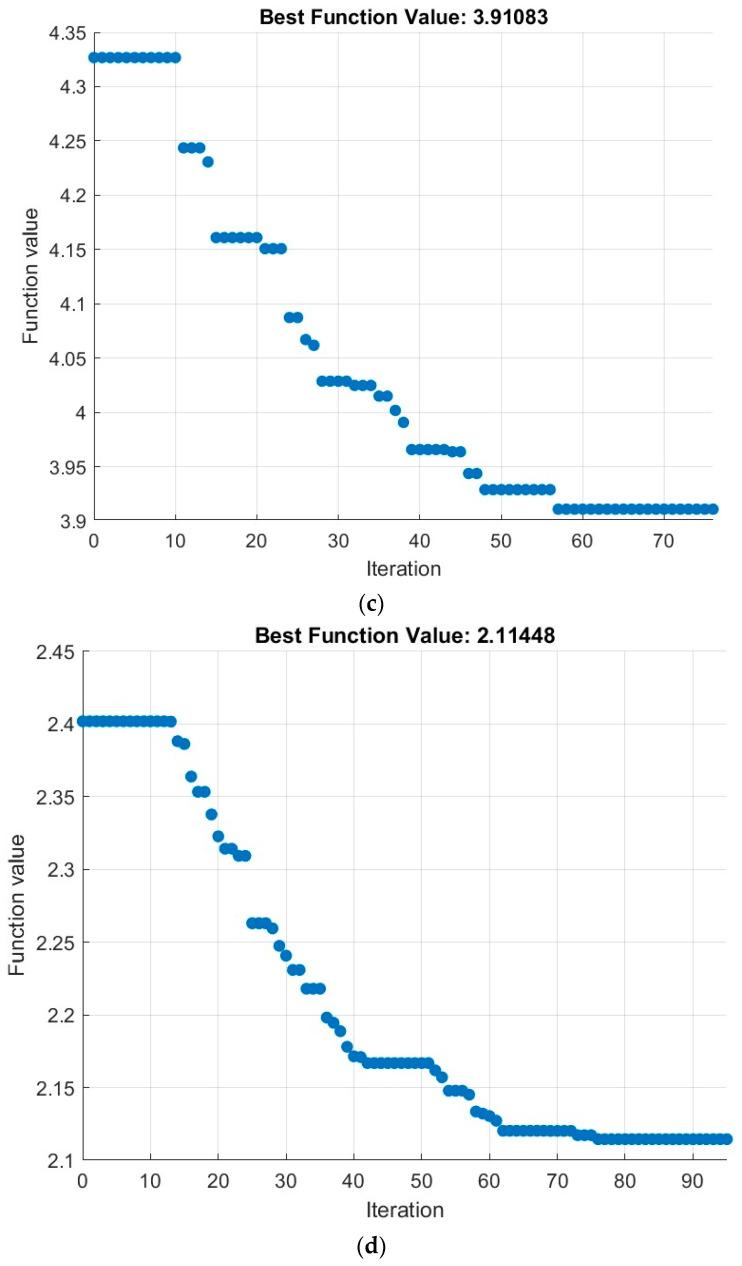

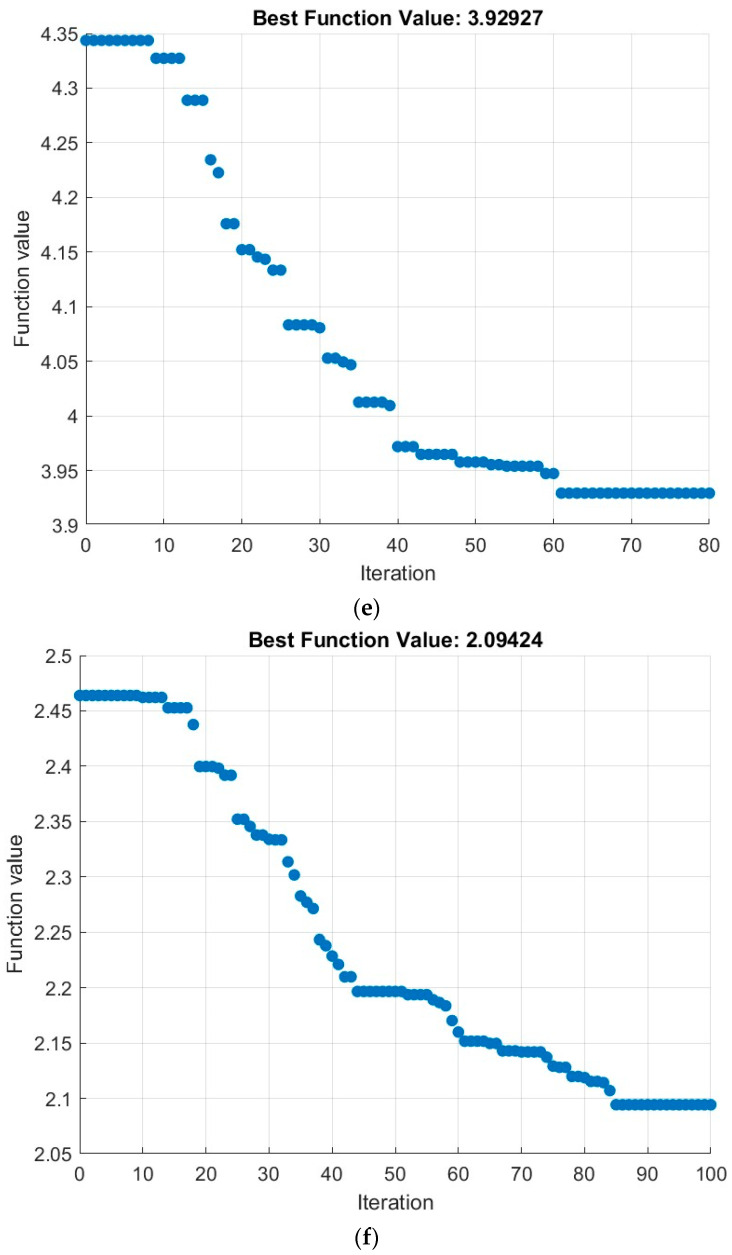
Fitness function values of the EPSO algorithm. (**a**) 50 sensor nodes at 3.6 GHz. (**b**) 100 sensor nodes at 3.6 GHz. (**c**) 50 sensor nodes at 26 GHz. (**d**) 100 sensor nodes at 26 GHz. (**e**) 50 sensor nodes at 38 GHz. (**f**) 100 sensor nodes at 38 GHz.

The EPSO algorithm is executed for multiple iterations until it reaches the maximum number of iterations or the algorithm converges to the best values of fitness function. Even though the maximum iterations are assigned as two hundred, the proposed algorithm converges at the early stage by itself, and this is proven by the results presented in Table 3, Table 4 and Table 5. The fitness function values converge within a smaller number of iterations at all frequencies and network sizes, as shown in Figure 5.

Some important observations were made from the simulation results: the convergence rate and coverage ratio of the proposed EPSO algorithm are higher than those of the existing [39] versions of PSO. Table 4 shows that the fitness function values are decreased with increasing network size. In addition, from Table 5, the number of detected holes is increased with the number of SNs; however, the size of these holes is minimized.

The major observations of the results presented in Table 3, Table 4 and Table 5 are that the fitness value decreases with increasing network size (in terms of the number of SNs). Simultaneously, the number of coverage holes is increased. However, the radius of the coverage holes was reduced. The EPSO method defines the communication range within 4 m, which avoids transmitting signals with high power. Therefore, an enhanced lifetime of the network is expected. At the same time, the communication radius of each SN is 80–120 m [32], 40 m [37], 30 m [35], and 6 m [33,39] which depletes the node’s battery faster. Additionally, the average number of iterations is 300 in [33], whereas the proposed EPSO algorithm provides early convergence within 100 iterations, and the average number of iterations is limited to 78, 82, and 80 at 3.6 GHz, 26 GHz, and 38 GHz, respectively.

In addition, the computational complexity of EPSO with DT is minimized to $O(S_{n}\log S_{n})$ compared with that of grid-based [22,37] deployments, whose complexity is $O(S_{n}^{2})$, where $S_{n}$ is the number of SNs. The EPSO algorithm provides higher coverage gains and a faster convergence rate than random deployments and grid-based [37] deployments of SNs. Overall, it was proven that the network coverage is enhanced using the proposed EPSO algorithm over the nascent variants of PSO algorithms [40,41,42].

## 5. Conclusions and Future Works

The deployment of sensor nodes in their optimal locations is highly important in monitoring, detecting, and tracking targets in next-generation wireless network applications. The random deployment of sensor nodes results in poor coverage in WSNs. To enhance the coverage area, an EPSO algorithm is proposed for computing the optimal node locations of sensor nodes. The EPSO algorithm avoids the deployment of sensor nodes in close proximity to each other and considers the battery lifetime at sensor nodes when addressing coverage holes. It detects the coverage holes in the initial deployment and ensures that every target is covered by at least one sensor node. The computational complexity of the EPSO algorithm is less because it generates ‘N’ one-dimensional swarms instead of N-dimensional swarms. The optimal inertia weights of the particles and acceleration factors are defined for optimizing the objective function and are applied when fine-tuning the sensor node positions. The simulation results of the EPSO algorithm validate its feasibility and yield converged results within a minimum number of iterations. The results of the sensor deployment and coverage showed that the required coverage conditions were met. However, the challenges in EPSO include the existence of dependencies among elements of different dimensions, which should be considered when executing this algorithm. Otherwise, the proposed algorithm may produce worse results than the conventional PSO algorithm. To overcome this problem, the dependent dimensions should be handled as a single swarm, which is the topic of future work.

## Figures and Tables

**Table 1:** Summary of existing node deployment and coverage algorithms in WSNs.

Ref. No.	Authors	Objective	Methodology	Remarks
[18]	Yarinezhad and Hashemi (2023)	Sensor deployment to cover targets and enhance network lifetime.	PSO algorithm, cooperative PSO using fuzzy logic. The fuzzy logic helped to dynamically compute acceleration coefficients.	The algorithm can be used for mobile targets. Network lifetime is increased.
[19]	Boualem et al. (2023)	The imperfections in random deployments of SNs are addressed.	A hybrid fuzzy-probabilistic model to schedule the passive or active state of SNs strategy is defined.	It dealt with the uncertainty of the SN’s deployment and infrastructure in terms of sensing, processing, and communication. It also addressed the uncertainty in cluster-head selection and maintained coverage between 99.99% and 90.00%.
[20]	Ammari, H.M. (2023)	The *k*-coverage problem in two-dimensional WSNs is addressed.	The optimal planar convex tile is determined to improve the sensing range usage. Hexagonal tiling-based approach is defined for sensor placement strategies. Relationships between the sensing and communication ranges are computed.	Network lifetime depends on the number of active SNs to cover ROI. There exists a correlation between the tiling problems and *k*-coverage.
[21]	Mahfouz et al. (2023)	The worst-case coverage for plane targets is proposed.	Clifford algebra, Voronoi diagram, and graph search algorithms are introduced for the coverage model.	The breach weight of the point target is less than the breach weight of the plane target. Only omnidirectional SNs are used.
[22]	Tarnaris et al. (2020)	To maximize the area coverage using computational intelligence algorithms.	Genetic algorithm (GA) and PSO algorithm are used.	PSO algorithm takes more execution time than GA. However, GA expects a larger population size. Voronoi-based and grid-based PSO algorithms enhance the coverage percentage.
[23]	Harizan and Kuila (2020)	Coverage and connectivity problems are addressed using metaheuristic algorithms.	GA, ant colony, optimization (ACO), and PSO are used.	ACO provides more low-cost and low-energy coverage solutions than other metaheuristic algorithms.
[24]	Chowdhury and De (2021)	Proposed an energy-efficient coverage optimization technique.	Voronoi-glowworm swarm optimization and K-means algorithm are used.	Optimal sensing radius is computed using Voronoi cell structure for efficient SN deployment. It improves the network lifetime with a sleep–wake mechanism. The coverage is 98% with an optimal number of active SNs.
[25]	Wang (2020)	To minimize the network delay by optimizing the path length between the gateway node and SN.	Adaptive PSO algorithm.	Introduced a random adjustment of inertia weights, adaptive neighborhood search, and learning factors.
[26]	Wang et al. (2023)	To optimize the SN coverage.	Self-adaptive multi-strategy artificial bee colony algorithm is used to enhance the global search capabilities.	It minimizes the SN redundancy and enhances the coverage rate by 14.1% over initial coverage values.
[27]	Abdallah et al. (2024)	It aims to enhance coverage rate with minimal SNs.	Delaunay triangulation and an artificial bee colony (DTABC) optimization algorithm is used.	Experiments are conducted at 2.4 GHz, the number of iterations is between 600 and 900, and transmission range is 10 m. The battery levels are discharged faster at the sensor nodes; typically, 1% is remaining in C-DTABC, and 8% is remaining in D-DTABC after 8000 s.
[28]	Kuang et al. (2024)	To optimize the sensing distance and sensing angle of acoustic sensors in underwater WSNs.	Adaptive heuristic algorithm.	It supports self-adjustment deployment, taking connectivity, coverage, and redundancy into account.
[29]	Ling et al. (2020)	To analyze coverage rate and network lifetime.	K-coverage model. Improved versions the of PSO algorithm.	Coverage that includes area coverage, target coverage, and boundary coverage are addressed. The required number of SNs is high, and it does not address mobile targets.
[30]	Zhang and Shen (2021)	To minimize the number of SNs and optimize their positions in the deployment.	Self-adaptive estimation PSO algorithm.	When the number of SNs is higher, the algorithm falls into local minima.
[31]	Zhao et al. (2022)	Coverage optimization.	PSO and chaos optimization algorithms.	PSO-circle improves coverage by 3.17% over the conventional PSO algorithm.
[32]	Chelbi et al. (2021)	To define optimal connectivity and coverage rate with a minimum number of SNs.	PSO and iterative local search algorithm.	Predefined potential positions for relay nodes deployment. Superior performance than canonical PSO, differential evolution, and GA.
[33]	Zhang (2020)	Coverage optimization.	Improved PSO algorithm and external dispersion method.	Overcomes the local convergence of PSO. Computational time and complexity are higher than PSO.
[34]	Li and Cao (2021)	Aiming at network coverage rate, coverage uniformity, and SN dormancy rate.	Discrete binary PSO algorithm.	Adaptive inertia weights and learning factor are introduced. The number of active SNs is minimal and enhances the network lifetime.
[35]	Wu et al. (2022)	To maximize network coverage rate.	Virtual force-directed PSO algorithm.	Global optimal is achieved by PSO. Virtual force fine-tuned by sensing probability for faster convergence.
[36]	Panag and Dhillon (2018)	To define the maximum number of disjoint-coverage sets of SNs for enhancing the network lifetime.	Random transition-based PSO.	It is suitable for both point coverage and area coverage applications.
[37]	Chowdhuri and Barma (2024)	To detect the coverage holes and mitigate them using deep learning techniques. To improve the reliability and comprehensiveness of monitoring by addressing the challenges due to coverage holes.	Improved social group optimization algorithm. Decentralized game optimization algorithm.	It reduces hole detection time by 87%–99.6%. It lowers time complexity by 26%–83%.
[38]	Simionato and Cimino (2024)	To detect and heal coverage holes.	A swarm intelligence-based algorithm.	Faster healing process from coverage holes.
[39]	Priyadarshi and Gupta (2023)	To maximize the coverage of area.	Modified PSO algorithm.	Negative velocity is introduced to avoid premature convergence.

**Table 2:** Simulation parameters.

Parameter	Value
Number of sensor nodes	10 to 100
Network terrain	100 m × 100 m
Communication radius of sensor node	4 m
Carrier frequency (n48, n258, n260 bands)	3.6 GHz, 26 GHz, 38 GHz
Max. iterations	200
Inertia weight of particle, w	0.85
Acceleration factors, $a_{1}$ and $a_{2}$	1.56, 1.56
Battery depletion threshold (absolute mean)	0.37 dBm

**Table 3:** Results of the proposed EPSO algorithm at 3.6 GHz band (n48 band).

Number of Nodes	Iteration No.	f-Count	Best f(x)	Mean f(x)	No. of Holes Detected
10	54	5500	18.92	20.25	8
15	34	3500	12.89	14.04	17
20	81	8200	9.686	10.27	20
25	51	5200	7.82	8.36	34
30	66	6700	6.459	6.838	38
35	44	4500	5.63	5.872	38
40	78	7900	4.874	5.049	43
45	100	10,100	4.381	4.602	59
50	87	8800	3.926	4.078	64
55	87	8800	3.59	3.748	66
60	69	7000	3.33	3.52	72
65	70	7100	3.109	3.258	76
70	85	8600	2.848	2.947	90
75	77	7800	2.727	2.823	96
80	100	10,100	2.536	2.665	91
85	100	10,100	2.391	2.497	105
90	100	10,100	2.335	2.467	106
95	97	9800	2.194	2.356	106
100	100	10,100	2.078	2.193	99

**Table 4:** Results of the proposed EPSO algorithm at 26 GHz (n258 band).

Number of Nodes	Iteration No.	f-Count	Best f(x)	Mean f(x)	No. of Holes Detected
10	34	3500	19.11	20.45	8
15	69	7000	12.73	13.57	16
20	58	5900	9.677	10.3	20
25	33	3400	7.822	8.323	28
30	85	8600	6.491	6.709	36
35	100	10,100	5.574	5.752	41
40	71	7200	4.907	5.141	53
45	100	10,100	4.418	4.642	61
50	76	7700	3.911	4.077	62
55	100	10,100	3.64	3.873	65
60	92	9300	3.335	3.537	70
65	76	7700	3.098	3.219	71
70	73	7400	2.891	3.035	80
75	87	8800	2.713	2.812	85
80	100	10,100	2.542	2.728	95
85	100	10,100	2.398	2.525	102
90	100	10,100	2.266	2.359	90
95	100	10,100	2.177	2.34	113
100	95	9600	2.114	2.205	110

**Table 5:** Results of the proposed EPSO algorithm at 38 GHz (n260 band).

Number of Nodes	Iteration No.	f-Count	Best f(x)	Mean f(x)	No. of Holes Detected
10	27	2800	19	20.3	8
15	36	3700	12.84	13.74	15
20	60	6100	9.668	10.03	24
25	46	4700	7.896	8.567	33
30	76	7700	6.46	6.726	39
35	100	10,100	5.545	5.744	42
40	68	6900	4.903	5.111	50
45	89	9000	4.4	4.569	56
50	80	8100	3.929	4.11	69
55	85	8600	3.607	3.807	65
60	100	10,100	3.348	3.511	69
65	94	9500	3.103	3.261	71
70	83	8400	2.937	3.066	90
75	100	10,100	2.683	2.828	82
80	83	8400	2.571	2.696	91
85	97	9800	2.392	2.501	90
90	100	10,100	2.303	2.371	102
95	100	10,100	2.19	2.288	109
100	100	10,100	2.094	2.23	119

## Data Availability

Data are shared upon reasonable request.

## References

[1] Srilakshmi A., Jyothsna V., Swaraja K., Dilli R. (2023). Gauss-Newton Multilateration Localization Algorithm in Large-scale Wireless Sensor Networks for IoT Applications. Telecommun. Radio Eng..

[2] Yang X., Luo C., Wang L., Liu H., Zhang L. (2024). Wireless optimisation positioning algorithm with the support of node deployment. Int. J. Comput. Sci. Eng..

[3] Meng J., Ning S. (2024). Application of intelligent algorithm in wireless sensor network node deployment optimization. J. Electron. Inf. Sci..

[4] Qi X., Li Z., Chen C., Liu L. (2022). A wireless sensor node deployment scheme based on embedded virtual force resampling particle swarm optimization algorithm. Appl. Intell..

[5] Kurian A.M., Onuorah M.J., Ammari H.M. (2024). Optimizing Coverage in Wireless Sensor Networks: A Binary Ant Colony Algorithm with Hill Climbing. Appl. Sci..

[6] Yang S.-Y., Xiang Y.-H., Kang D.-W., Zhou K.-Q. (2024). An Improved Cuckoo Search Algorithm for Maximizing the Coverage Range of Wireless Sensor Networks. Baghdad Sci. J..

[7] Xie M., Pi D., Dai C., Xu Y. (2024). A metaheuristic-based algorithm for optimizing node deployment in wireless sensor network. Neural Comput. Appl..

[8] Ben Amor O., Chelly Dagdia Z., Bechikh S., Ben Said L. (2024). Many-objective optimization of wireless sensor network deployment. Evol. Intell..

[9] Kusuma S.M., Veena K.N., Kumar B.P.V., Naresh N., Marianne L.A. (2024). Meta Heuristic Technique with Reinforcement Learning for Node Deployment in Wireless Sensor Networks. SN Comput. Sci..

[10] Boualem A., De Runz C., Kholidy H., Bengheni A., Taibi D., Ayaida M., Yang X.S., Sherratt S., Dey N., Joshi A. (2024). A New Classification of Target Coverage Models in WSNs, Survey and Algorithms and Future Directions. Lecture Notes in Networks and Systems, Proceedings of Ninth International Congress on Information and Communication Technology, ICICT 2024, London, UK, 19–22 February 2024.

[11] Sahal N., Murugan R., Raizada A. Deployment Strategies for Wireless Sensor Networks Using Math Modelling Techniques. Proceedings of the 2024 International Conference on Optimization Computing and Wireless Communication (ICOCWC).

[12] Binh H.T.T., Hanh N.T., Tan N.P., Quan L.V., Ngoc D.T., Minh N.H.N., Phap H.C. (2024). A heuristic node placement strategy for extending network lifetime and ensuring target coverage in mobile wireless sensor networks. Evol. Intell..

[13] Jaiswal K., Anand V. (2024). ESND-FA: An Energy-Efficient Scheduled Based Node Deployment Approach Using Firefly Algorithm for Target Coverage in Wireless Sensor Networks. Int. J. Wirel. Inf. Netw..

[14] Jia R., Zhang H. (2024). Wireless Sensor Network (WSN) Model Targeting Energy Efficient Wireless Sensor Networks Node Coverage. IEEE Access.

[15] Layolin Benisto L.C., Sukumaran R., Kumar A.S.C. Node Deployment Strategies and Challenges in Underwater Wireless Sensor Network. Proceedings of the 2024 5th International Conference on Mobile Computing and Sustainable Informatics (ICMCSI).

[16] Lu L., Mao Y. (2024). Coverage Optimization Strategy of UWSNs Based on Improved Sparrow Search Algorithm. Acad. J. Sci. Technol..

[17] Wang J., Zhu Z., Zhang F., Liu Y. (2024). An improved salp swarm algorithm for solving node coverage optimization problem in WSN. Peer-to-Peer Netw. Appl..

[18] Yarinezhad R., Hashemi S.N. (2023). A sensor deployment approach for target coverage problem in wireless sensor networks. J. Ambient. Intell. Humaniz. Comput..

[19] Boualem A., De Runz C., Ayaida M., Akdag H. (2023). A fuzzy/possibility approach for area coverage in wireless sensor networks. Soft Comput..

[20] Ammari H.M. (2023). A Computational Geometry-based Approach for Planar k-Coverage in Wireless Sensor Networks. ACM Trans. Sens. Netw..

[21] Mahfouz A.M., Ismail A.S., El Sobky W.I., Nasry H. (2023). A novel model for representing a plane target and finding the worst-case coverage in wireless sensor network based on Clifford algebra. J. Wirel. Commun. Netw..

[22] Tarnaris K., Preka I., Kandris D., Alexandridis A. (2020). Coverage and k-Coverage Optimization in Wireless Sensor Networks Using Computational Intelligence Methods: A Comparative Study. Electronics.

[23] Harizan S., Kuila P., Das S., Samanta S., Dey N., Kumar R. (2020). Evolutionary Algorithms for Coverage and Connectivity Problems in Wireless Sensor Networks: A Study. Design Frameworks for Wireless Networks.

[24] Chowdhury A., De D. (2021). Energy-efficient coverage optimization in wireless sensor networks based on Voronoi-Glow worm Swarm Optimization-K-means algorithm. Ad Hoc Netw..

[25] Wang W. (2020). Deployment and optimization of wireless network node deployment and optimization in smart cities. Comput. Commun..

[26] Wang J., Liu Y., Rao S., Zhou X., Hu J. (2023). A novel self-adaptive multi-strategy artificial bee colony algorithm for coverage optimization in wireless sensor networks. Ad Hoc Netw..

[27] Abdallah W., Mnasri S., Val T. (2024). Centralized and distributed approaches of Artificial Bee Colony algorithm and Delaunay Triangulation for the coverage in IoT networks. Peer-to-Peer Netw. Appl..

[28] Kuang Y., Jiang B., Cui X., Li S., Wang J., Song H. (2024). Adaptive double-loop coverage optimization of underwater wireless directional restricted sensor networks. Ad Hoc Netw..

[29] Ling H., Zhu T., He W., Luo H., Wang Q., Jiang Y. (2020). Coverage Optimization of Sensors under Multiple Constraints Using the Improved PSO Algorithm. Math. Probl. Eng..

[30] Zhang Y., Shen W. A Novel Particle Swarm Optimization Algorithm for k-Coverage Problems in Wireless Sensor Networks. Proceedings of the 2021 IEEE 24th International Conference on Computer Supported Cooperative Work in Design (CSCWD).

[31] Zhao Q., Li C., Zhu D., Xie C. (2022). Coverage Optimization of Wireless Sensor Networks Using Combinations of PSO and Chaos Optimization. Electronics.

[32] Chelbi S., Dhahri H., Bouaziz R. (2021). Node placement optimization using particle swarm optimization and iterated local search algorithm in wireless sensor networks. Int. J. Commun. Syst..

[33] Zhang Y. (2020). Coverage Optimization and Simulation of Wireless Sensor Networks Based on Particle Swarm Optimization. Int. J. Wirel. Inf. Netw..

[34] Li Y., Cao J. (2021). WSN node optimal deployment algorithm based on adaptive binary particle swarm optimization. ASP Trans. Internet Things.

[35] Wu L., Qu J., Shi H., Li P. (2022). Node Deployment Optimization for Wireless Sensor Networks Based on Virtual Force-Directed Particle Swarm Optimization Algorithm and Evidence Theory. Entropy.

[36] Panag T.S., Dhillon J.S. (2018). A Novel Random Transition Based PSO Algorithm to Maximize the Lifetime of Wireless Sensor Networks. Wirel. Pers. Commun..

[37] Chowdhuri R., Barma M.K.D. (2024). Enhancing Network Reliability: Exploring Effective Strategies for Coverage-Hole Analysis and Patching in Wireless Sensor Networks. Wirel. Pers. Commun..

[38] Simionato G., Cimino M.G.C.A. (2024). Swarm intelligence for hole detection and healing in wireless sensor networks. Comput. Netw..

[39] Priyadarshi R., Gupta B. (2023). 2-D coverage optimization in obstacle-based FOI in WSN using modified PSO. J. Supercomput..

[40] Jubair A.M., Hassan R., Aman A.H.M., Sallehudin H. (2021). Social class particle swarm optimization for variable-length Wireless Sensor Network Deployment. Appl. Soft Comput..

[41] Shen J., Zhu D., Li R., Zhu X., Zhang Y., Li W., Zhou C., Zhang J., Cheng S. (2024). Efficient base station deployment in specialized regions with splitting particle swarm optimization algorithm. World Wide Web.

[42] Kim J.S., Lee D.H., Yanf W., Kim Y.S., Choi J.W., Kwon H., Park J., Son S.-U., Bae H.S., Park J.-S. (2024). Optimal deployment of bistatic sonar using particle swarm optimization algorithm. J. Acoust. Soc. Korea.

[43] Dilli R. Analysis of 5G Wireless Systems in FR1 and FR2 Frequency Bands. Proceedings of the 2020 2nd International Conference on Innovative Mechanisms for Industry Applications (ICIMIA).

